# COVID-19: Up to 82% critically ill patients had low Vitamin C values

**DOI:** 10.1186/s12937-021-00727-z

**Published:** 2021-07-09

**Authors:** Teresa Maria Tomasa-Irriguible, Lara Bielsa-Berrocal

**Affiliations:** Intensive Care Department, Germ, ans Trias I Pujol Hospital, Germans Trias I Pujol University Hospital, Autónoma de Barcelona University, Carretera del Canyet s/n, 08916 Badalona, , Barcelona Spain

**Keywords:** COVID-19, SARS-CoV-2, Vitamin C, ARDS, ICU, Critically ill patient, Epidemiology

## Abstract

There are limited proven therapeutic options for the prevention and treatment of COVID-19. We underwent an observational study with the aim of measure plasma vitamin C levels in a population of critically ill COVID-19 adult patients who met ARDS criteria according to the Berlin definition. This epidemiological study brings to light that up to 82% had low Vitamin C values. Notwithstanding the limitation that this is a single-center study, it nevertheless shows an important issue. Given the potential role of vitamin C in sepsis and ARDS, there is gathering interest of whether supplementation could be beneficial in COVID-19.

## Main text

There are limited proven therapeutic options for the prevention and treatment of COVID-19. Given the potential role of vitamin C in sepsis and ARDS, there is gathering interest of whether supplementation could be beneficial in COVD-19 [1, 2]. However, first of all, we have to know if the incidence of vitamin C deficiency is high.

We underwent an observational study with the aim of measure plasma vitamin C levels in a population of critically ill COVID-19 adult patients who met ARDS criteria according to the Berlin definition [3]. The patients were admitted to hospital a median 10 days after onset of symptoms and the last intake was the day before hospital admission. We do not know if due to COVID-19 the usual food intake was modified and, therefore, the vitamin C status was affected. The truth is that the overall intake had been reduced by half in most cases.

Vitamin C levels were measured within the first 24 h of ICU admission. The admission took place in ICU since first moment of hospitalization. The study was approved by the local Clinical Research Ethics Committee (PI-20–253).

We included 67 patients from Mars to April. Median age was 60 and 75% were male. The need of intubation was high (73%) and more than 80% of them require prone position. Acute kidney Injury was registered in more than 30%, however only 7% require renal replacement therapies. In-hospital mortality was around 22%. Main characteristics of the population included are presented in Table 1.

**Table 1:** Clinical characteristics of the COVID-19 patients included. Results of continuous variables are expressed as median and minimum and maximum. Categorical variables are expressed as frequency (percentage). SOFA sequential organ failure assessment, ICU intensive care unit, ECMO extracorporeal membrane oxygenation, AKI acute kidney injury, CRRT continuous renal replacement therapy, IRR intermittent renal replacement, CKD chronic kidney disease, ICU-LOS Length of ICU stay, HOSP-LOS Length of hospital stay

Clinical characteristics	COVID-19 with ARDS (n = 67)
Age (median, min–max, years)	60 (31 – 76)
Male (n, %)	50 (75)
SOFA score (median, min–max, points)	9 (2 – 17)
Intubation (n, %)	49 (73.1)
Prone position (n, % respect of intubation)	40 (81.6)
VV-ECMO (n, %)	3 (4.5)
VA-ECMO (n, %)	1 (1.5)
Noradrenaline (n, %)	40 (59.7)
Dobutamine (n, %)	6 (9)
CKD (n, %)	6 (9)
AKI (n, %)	22 (32.8)
CRRT (n, %)	3 (4.5)
IRR (n, %)	2 (3)
Bacterial superinfection (n, %)	23 (34.3)
ICU-LOS (median, min–max, days)	12 (1 -90)
HOSP-LOS (median, min–max, days)	25 (1 – 101)
Mortality (n, %)	15 (22.4)

Vitamin C was determined by high-performance liquid chromatography (HPLC). Procedure: Once the blood sample was drawn, it was quickly protected from light and processed with maximum speed. Lab sequence: 1) separating the plasma (EDTA) in a 10 min, 3000 rev and 14ºC centrifugation; 2) pipetting 1 ml of centrifuged plasma into the supplied tube (1 ml homocysteine 0.7 mg/mL in 10% trichloroacetic acid) and mixing it repeatedly; 3) centrifuging again for 10 min at 3000 rev and 14ºC; and finally 4) separating and freezing the supernatant immediately at -80ºC, which was sent to the central laboratory. Vitamin C was determined by high-performance liquid chromatography.

The range of normality of vitamin C plasma values are 0.4–2 mg/dL. Up to 82% had low Vitamin C values. The median value of vitamin C was 0.14 mg/dL with a minimum value of < 0.10 mg/dL and a maximum of 1.08 mg/dL; the mean value was 0.14 mg/dL and standard deviation was 0.05 mg/dL. There were 12 patients with a value below 0.10 mg/dL. Main results are shown in Table 2.

**Table 2:** Vitamin C results in COVID-19 patients. Values are expressed as mean and standard deviation. Categorical values are expressed as frequency (percentage)

Vitamin C status	n = 67
Low values (0.1–0.4 mg/dL)	
N (%)	43 (64.2)
Mean (SD)	0.14 (0.05)
Undetectable values (< 0.1 mg/dL)	
N (%)	12 (17.9)
Low & Undetectable values	
N (%)	55 (82)
Normal values (normal range: 0.4–2 mg/dL)	
N (%)	12 (17.9)
Mean (SD)	0.59 (0.18)

## Discussion

Vitamin C is an essential water-soluble nutrient, required as a cofactor for a number of enzymatic reactions [4]. Its effects on the immune system during infection include the promotion of phagocytosis and chemotaxis of leucocytes and development and maturation of T-lymphocytes. Animal studies support a beneficial role of vitamin C. These positive effects include increased resistance of chick embryo tracheal organ cultures to infection and protecting broiler chicks against avian coronavirus [5, 6]. Vitamin C had been shown to significantly decrease serum TNFα and IL-1β levels and increased superoxide dismutase, catalase, and glutathione levels in a rat ARDS model supporting its antioxidant effect [7]. Additionally, vitamin C also enhances lung epithelial barrier function by promoting epigenetic and transcriptional expression of protein channels at the alveolar capillary membrane that regulate alveolar fluid clearance, which include aquaporin-5, cystic fibrosis transmembrane conductance regulator, epithelial sodium channel, and the Na + /K + -ATPase pump [8].

The present study has several limitations. It is an observational study conducted in a single center with small sample size of critically ill patients. We found almost 18% of undetectable Vitamin C values. However, vitamin C status could be affected by various confounding factors. As we know, Vitamin C is very sensitive to light and to oxidation, so that once the blood sample is drawn, it must quickly be protected from light and processed with maximum speed. It is possible that we had some trouble few vitamin C blood samples before processed. We would like to address this issue because it is important. The recently published study by Chiscano-Camón et al. [9] described a 94.4% undetectable vitamin C levels for COVID-19 patients. This data caught our attention. They included 18 patients with severe acute respiratory syndrome coronavirus-2 (SARS-CoV-2) disease and Vitamin C was also determined by high-performance liquid chromatography with photodiode detector. They hypothesized that several mechanisms may be involved, such as increased metabolic consumption due to the enhanced inflammatory response, glomerular hyperfiltration, dialysis, decreased gastrointestinal absorption, or decreased recycling of dehydroascorbate to ascorbic acid. Nevertheless, we think it is important to be aware with the fact that there might be a problem with blood samples and the measurement method.

Nonetheless, our study shows that most critically ill COVID-19 patients have low Vitamin C values. Research is ongoing with COVID-19 disease with a high-dose intravenous vitamin C (HDIVC) with a clinical trial investigating vitamin C infusion for severe 2019-nCoV-infected pneumonia in Wuhan, China [10].

## Conclusion

In conclusion, in our cohort of patients with COVID-19-associated ARDS, vitamin C status is very low. Further studies are needed to find out real incidence of vitamin C deficiency and if treatment with vitamin C has any impact COVID-19 disease.

## Data Availability

Not Applicable.

## Abbreviations

SARS-CoV-2: Name of this novel coronavirus
COVID-19: Disease caused by SARS-CoV-2
ARDS: Acute Respiratory Distress Syndrome
ICU: Intensive Care Unit
SOFA: Sequential organ failure assessment
ECMO: Extracorporeal membrane oxygenation
AKI: Acute kidney injury
CRRT: Continuous renal replacement therapy
IRR: Intermittent renal replacement
CKD: Chronic kidney disease
ICU-LOS: Length of ICU stay
HOSP-LOS: Length of hospital stay
